# Cardiac CT Roadmap for Left Atrial Appendage Occlusion: From Planning to Strategy to Follow-Up

**DOI:** 10.3390/jcm15197361

**Published:** 2026-09-22

**Authors:** Antonella Meloni, Katia Capellini, Benigno Marco Fanni, Emanuele Gasparotti, Rossana Buongiorno, Ilaria Verdirame, Lorenza Petrini, Giancarlo Pennati, Francesca Berti, Vincenzo Positano, Guido Parodi, Giovanni Benedetti, Luigi Emilio Pastormerlo, Sergio Berti, Simona Celi

**Affiliations:** 1 Bioengineering Unit, Fondazione Monasterio, 56124 Pisa, Italy; antonella.meloni@ftgm.it (A.M.); positano@ftgm.it (V.P.); 2 BioCardioLab, Bioengineering Unit, Fondazione Monasterio, 54100 Massa, Italy; kcapellini@ftgm.it (K.C.); bmfanni@ftgm.it (B.M.F.); gasparotti@ftgm.it (E.G.); rbuongiorno@ftgm.it (R.B.); iverdirame@ftgm.it (I.V.); 3 Department of Information Engineering, University of Pisa, 56122 Pisa, Italy; 4 Department of Civil and Environmental Engineering, Politecnico di Milano, 20133 Milan, Italy; lorenza.petrini@polimi.it; 5 Laboratory of Biological Structure Mechanics, Department of Chemistry, Materials and Chemical Engineering “Giulio Natta”, Politecnico di Milano, 20133 Milan, Italy; giancarlo.pennati@polimi.it (G.P.); francesca.berti@polimi.it (F.B.); 6 Cardiology Unit, Department of Medicine, Lavagna Hospital, 16033 Lavagna, Italy; guido.parodi@asl4.liguria.it; 7 Diagnostic and Interventional Cardiology Unit, Fondazione Monasterio, 54100 Massa, Italy; benedetti@ftgm.it (G.B.); pastormerlo@ftgm.it (L.E.P.); berti@ftgm.it (S.B.)

**Keywords:** left atrial appendage occlusion, cardiac computed tomography, planning

## Abstract

Cardiac computed tomography (CCT) has emerged as a central imaging modality in the workflow for left atrial appendage occlusion (LAAO), as accurate anatomical assessment is vital for successful intervention. CCT provides superior three-dimensional (3D) anatomical detail and isotropic spatial resolution compared to traditional trans-esophageal echocardiography, enabling precise pre-procedural planning, device sizing, and identification of optimal fluoroscopic projections. Furthermore, CCT serves as a highly accurate, non-invasive tool for detecting post-procedural complications such as peri-device leaks and device-related thrombosis. Beyond conventional imaging, CCT-derived datasets now facilitate advanced computational applications, including patient-specific 3D printing, computational fluid dynamics, and finite element modeling, to predict device-tissue interactions and post-implant hemodynamics. Additionally, artificial intelligence is increasingly integrated to automate segmentation and enhance risk stratification. This review provides a comprehensive overview of the expanding role of CCT in LAAO, highlighting its contribution to procedural optimization and the transition toward personalized, engineering-driven cardiac interventions.

## 1. Introduction

Atrial fibrillation (AF) is the most prevalent sustained cardiac arrhythmia. AF is a major contributor to thromboembolic events, particularly ischemic stroke, which represents a substantial global burden of morbidity and mortality [1,2,3]. In patients with non-valvular AF, the left atrial appendage (LAA) is the primary site of thrombus formation because of its marked anatomical variability, structural complexity, and impaired contractility, which promote blood stasis [3,4]. The risk of thromboembolic events is routinely estimated using the CHA_2_DS_2_-VASc score, which incorporates age, sex, and major cardiovascular comorbidities [5]. Current guidelines recommend oral anticoagulation for patients at increased thromboembolic risk, particularly those with a CHA_2_DS_2_-VASc score ≥ 2 [6]. However, long-term anticoagulation is frequently limited by bleeding risk, contraindications, or poor drug tolerance, leaving a substantial proportion of patients at persistently high risk of stroke [7,8]. Percutaneous left atrial appendage occlusion (LAAO) has emerged as a validated alternative to anticoagulants for stroke prevention in selected patients at moderate-to-high thromboembolic risk who are unsuitable for long-term anticoagulation [9,10], as supported by contemporary evidence and current guidelines [6,11].

LAAO is a minimally invasive procedure designed to mechanically exclude the LAA from the systemic circulation. The procedure is performed via the femoral vein and requires transseptal puncture (TSP) at the fossa ovalis (FO) to access the left atrium, through which an occluding device is delivered using a dedicated sheath or catheter. Even if percutaneous LAAO is minimally invasive and warrants fast post-procedural recovery [12], the procedure remains technically challenging due to the extensive inter-individual variability of the LAA anatomy [13]. Moreover, to offer superior clinical outcomes, LAAO operators need rigorous multi-stage imaging protocols, integrating pre-procedural planning, real-time intra-procedural guidance, and longitudinal post-procedural surveillance. Although serious procedural complications remain uncommon [14], life-threatening events such as LAA or cardiac perforation causing pericardial tamponade or unretrievable device embolization may occasionally require emergent cardiac surgery (ECS). In historical LAAO trials, surgical conversion rates of up to 1.5% were reported [15]. With advances in device technology, patient selection, procedural techniques, and operator experience, the need for surgical bailout has progressively decreased [16]. In contemporary LAAO practice, ECS has become an extremely rare event, with reported rates of approximately 0.1–0.3% in large registries, including the NCDR LAAO, EWOLUTION, and EMERGE LAA studies [17,18,19].

Typically, trans-esophageal echocardiography (TEE) has been the reference imaging modality for procedural planning and intra-procedural guidance [6,20]. However, its two-dimensional nature and operator dependence may limit accurate assessment of complex or multilobed anatomies [21,22]. Moreover, TEE often does not give a complete evaluation of the spatial relation between fossa ovalis, left atrium and LAA.

Cardiac computed tomography (CCT) has progressively evolved into a central imaging modality in the LAAO workflow. By offering superior three-dimensional (3D) anatomic detail and isotropic spatial resolution compared with TEE, CCT provides detailed anatomical information on the LAA, including its morphology, ostium dimension, and spatial relationship with adjacent structures, all of which are critical for accurate device selection and procedural strategy [3,21,23]. Furthermore, CCT plays an important role in post-procedural assessment, enabling the detection of device-related complications, such as peri-device leakage (PDL) and device-related thrombosis (DRT), and supporting follow-up evaluation [24]. More recently, CCT has become central to advanced computational applications in LAAO, including flow simulations and device modeling to better understand post-implant hemodynamics [25]. In parallel, artificial intelligence (AI) supports automated analysis and personalized procedural planning [26,27].

This narrative review aims to provide a structured and comprehensive overview of the expanding role of CCT in LAAO, encompassing (i) diagnosis and anatomical assessment, (ii) procedural planning, (iii) post-procedural evaluation, and (iv) emerging computational and experimental technologies. Specific attention is given to the contributions to procedural optimization and future directions in personalized cardiac intervention. Figure 1 depicts an overview of the main topics covered by this review.

## 2. Diagnostic and Morphological Analysis of LAA via CCT

CCT has become an essential tool in clinical practice for both LAA morphological characterization and detection of intracardiac thrombi [28,29].

### 2.1. LAA Anatomy and Morphological Classification

Electrocardiogram (ECG)-gated CCT provides isotropic high-resolution 3D datasets that enable detailed visualization of LAA geometry, including spatial orientation, trabecular architecture, and distal lobar complexity [30], overcoming the inherent limitations of two-dimensional imaging, which is constrained by acoustic windows [31].

By combing CT and magnetic resonance imaging (MRI) scans, Di Biase et al. proposed a 3D classification of LAA morphologies [28]. Four main variants were identified: “chicken wing”, “cactus”, “windsock”, and “cauliflower”. According to this classification, the main structures for these four types are characterized as follows:

- Chicken wing (CW): a single dominant lobe with an early, sharp bend along its length;
- Cactus (CC): a dominant central lobe with multiple secondary lobes branching out superiorly and inferiorly;
- Windsock (WS): a long, dominant central lobe with one or more secondary lobes;
- Cauliflower (CF): an irregular, highly trabeculated LAA lacking a distinctly dominant lobe.

The incidence of each shape is 48%, 30%, 19%, and 3%, respectively [28]. The “chicken wing” morphology has been consistently associated with a lower risk of embolic stroke compared with the other morphologies [32]. Figure 2 depicts a CCT slice with the relative 3D reconstruction for each LAA morphology, with the associated risk of thrombus, according to Di Biase et al. [28].

By leveraging high-resolution volumetric datasets, CCT enables the precise measurement of anatomical markers such as ostial eccentricity and structural irregularity, which are critical for thromboembolic risk stratification. Specifically, large secondary lobes with a base orifice diameter exceeding 10 mm increase structural complexity and are associated with embolic stroke of undetermined source (ESUS) [33], while increased left atrial wall thickness has been identified as an independent CCT-derived predictor of cardioembolic stroke [34].

Advanced computational approaches applied to CT imaging, such as fractal dimension analysis using the box-counting method, allow objective quantification of morphological irregularity and improve thrombosis prediction, particularly in low- to intermediate-risk patients, where CHA_2_DS_2_-VASc may be less discriminative [35].

LAA morphology undergoes progressive remodeling driven by aging and the duration of AF, resulting in a decreased prevalence of the “chicken wing” phenotype [36]. Beyond static evaluation, multiphase four-dimensional (4D) ECG-gated CCT facilitates a dynamic assessment of the LAA throughout the cardiac cycle. This advanced approach enables the non-invasive quantification of functional parameters, including ostial area variation, centerline tortuosity, and the LAA ejection fraction (LAA-EF) [37,38]. Figure 3 shows examples of the four LAA variants in terms of morphology and ostium areas along the different phases of the cardiac cycle.

By analyzing the time-variant morphologies of the LAAs, it has been observed that both “windsock” and “cauliflower” are characterized by wider orifices and lower tortuosity variation, promoting blood stasis [39,40]. In contrast, the “chicken wing” morphology exhibits more effective washout dynamics, which may also influence thrombus composition, with lower fibrin content observed in these patients [41]. Overall, by integrating anatomical and functional parameters, 4D CCT provides a comprehensive morpho-functional characterization of the LAA that significantly enhances thromboembolic risk stratification.

### 2.2. Thrombus Detection

The formation of in situ thrombi represents the ultimate consequence of LAA morphological complexity and blood stasis.

CCT has emerged as a robust, non-invasive alternative to TEE for LAA thrombus detection, demonstrating high diagnostic performance and a very high negative predictive value for the exclusion of thrombus [42,43]. However, the main diagnostic limitation of standard early-phase CT is the differentiation between true thrombus and circulatory stasis (spontaneous echo contrast or “sludge”), as both appear as hypoattenuating filling defects [44]. To address this issue, delayed-phase imaging has become a critical component of optimized CCT protocols. By acquiring additional scans after contrast administration, it is possible to distinguish transient filling defects due to slow flow, which progressively opacify, from true thrombi, which remain persistently unenhanced. This approach significantly improves diagnostic specificity and overall accuracy. In particular, delayed imaging protocols have been shown to increase sensitivity and specificity up to 100% and 99%, respectively, markedly enhancing positive predictive value and reducing false-positive findings [45]. Despite its high performance, the optimal timing of delayed acquisition remains a matter of debate and may vary according to individual hemodynamics, contrast administration protocols, and technical factors. Different strategies have been proposed, including 1, 3, and 6 min delayed scans, all demonstrating improved diagnostic yield compared with single-phase imaging [43,45]. In selected cases with persistent indeterminate findings, additional techniques such as prone positioning may further aid differentiation by promoting contrast redistribution within the LAA, although these approaches are not routinely adopted due to logistical constraints. From a practical perspective, CCT-based workflows can be tailored to minimize radiation exposure: when no filling defect is observed during the early phase, delayed acquisitions may be safely omitted, reducing unnecessary radiation and contrast administration [43]. Conversely, multiphase protocols or dual-enhanced techniques may be considered in equivocal cases, although the latter may increase contrast load and limit applicability in patients with renal impairment. Overall, when optimized acquisition protocols are implemented, CCT serves as a highly accurate, reproducible gatekeeper that streamlines pre-procedural assessment and minimizes patient risk by leveraging its exceptional negative predictive value to exclude LAA thrombi.

## 3. CT-Based Pre-Procedural Planning for LAA Occlusion

Pre-procedural imaging is a cornerstone of LAAO, directly influencing procedural safety, device selection, and clinical outcomes. Its primary objectives include exclusion of LAA thrombus, detailed characterization of appendage anatomy for accurate device sizing, assessment of adjacent cardiac structures, and identification of optimal fluoroscopic projections and TSP strategy [46,47].

CCT is increasingly adopted as a non-invasive alternative to TEE, supporting a workflow where trans-esophageal or intracardiac echocardiography are reserved for intraprocedural guidance [47,48]. Although CCT may offer lower temporal resolution than TEE, it compensates by providing a comprehensive 3D assessment of the left atrium and LAA, which is particularly advantageous for evaluating anatomically complex or multilobed appendages [48,49].

Standard CCT protocols for LAA evaluation are performed on scanners with at least 64 detector rows and typically follow a biphasic acquisition strategy [47,48]. The initial phase consists of an ECG-gated scan with submillimeter spatial resolution, usually acquired in systole (approximately 30–60% of the R–R interval), and provides the primary morphological dataset for procedural planning, including assessment of appendage anatomy and sizing. ECG gating can be performed using either a prospective or retrospective technique: prospective gating reduces radiation exposure but is more susceptible to arrhythmias and does not allow multiphase reconstruction, whereas retrospective gating enables flexible post-processing at the expense of a higher radiation dose. This is followed by a delayed acquisition, which is specifically used to differentiate slow contrast opacification or blood stasis from true filling defects such as thrombus.

Beyond simple dimensional measurements, CCT enables evaluation of ostial shape and eccentricity, non-planar geometry, axis orientation, lobar configuration, and depth-to-orifice relationships, all of which influence device stability and sealing performance [50,51,52]. Because the LAA ostium is frequently elliptical and irregular [53], multiplanar reconstruction (MPR) is essential to obtain true orthogonal measurements along the appendage axis and reduce underestimation of maximal diameters [50]. In addition to maximal and minimal diameters, CCT allows assessment of area-derived parameters that may better reflect the true landing zone geometry [54]. Evaluation of the landing zone also includes local curvature, wall tapering, trabecular support, and depth-to-diameter relationships, which are important determinants of anchoring stability and complete sealing [51]. Similarly, accurate multiplanar identification of the FO is an important component of preprocedural planning. Assessment of its anatomical position and spatial relationship with the LAA enables prediction of the TSP site and definition of the LAAO feasibility [55]. Figure 4 illustrates the representative MPR CCT reconstruction planes used for identification of FO and for defining the LAA ostial plane, using the left circumflex coronary artery (LCX) as the inferior anatomical landmark [56].

By assessing the spatial relationship between the LAA and the interatrial septum, CCT enables the precise identification of the ideal TSP site, typically located in the posteroinferior septum, which is critical for achieving coaxial alignment of the LAAO delivery system with the main axis of the appendage [49,55]. This preprocedural planning allows operators to determine optimal fluoroscopic imaging angles and optimized on-axis device deployment, thereby minimizing the risk of PDL and reducing the need for multiple device repositioning or the use of multiple closure devices [57].

Moreover, thanks to its large field of view, CCT allows for a comprehensive evaluation of surrounding anatomical structures, including the LCX, pulmonary artery, left superior pulmonary vein (LSPV), and mitral valve [51,58]. This is particularly relevant in anatomies where the appendage is closely related to surrounding structures and where device implantation may increase the risk of mechanical interaction or impingement [59,60,61].

### 3.1. Device Selection and Sizing

Device-specific planning is required because each occlusion system relies on different definitions of the ostium and landing zone and each has a different sizing chart [62].

Currently available percutaneous LAAO devices are based on two main principles:

- Plug: the device has a lobe or umbrella directly obstructing the neck of the LAA;
- Pacifier: the device comprises a lobe or umbrella part and an additional disc to seal the LAA ostium from the left atrial side.

Currently, the two most common and most studied devices used in clinical practice are the Watchman FLX^®^ and the Amplatzer Amulet^®^. Figure 5a shows a representative LAA with indication of the main measurements required to select the sizing for Watchman FLX and Amplatzer Amulet occluders. The Watchman FLX is a plug-type, self-expanding nitinol device equipped with fixation hooks with a dual-row anchor configuration, to support anchoring and device stability. A permeable polyethylene terephthalate (PET) fabric covers the atrial surface, facilitating appendage sealing (Figure 5b) [63]. The Amplatzer Amulet is the most common pacifier device, a self-expanding nitinol device. It is characterized by a dual seal system reinforced by two distal and proximal PET patches. Anchors on a single row are present in the distal lobe to guarantee the fixation to the LAA tissue (Figure 5c) [3,64,65]. For the Watchman FLX, sizing is determined by the anatomical ostium, defined between the circumflex artery inferiorly and a point 1–2 cm within the LSPV ridge, using the maximum diameter in this plane with slight oversizing. Conversely, the Amplatzer Amulet requires a dual-parameter evaluation of both the ostium and a more distal landing zone (approximately 10 mm and 12–15 mm within the appendage, respectively) to accommodate the lobe-and-disc architecture. Finally, LAA depth must be assessed from the ostial center to the main lobe apex in sagittal and coronal views to ensure stable implantation and successful closure. In both systems, accurate assessment of appendage depth is essential to ensure stable implantation and avoid device protrusion or incomplete closure.

Due to the complexity of the LAA, the ostium size as well as the landing zone and the depth of the LAA are assessed on MPR CCT, which may sometimes require maximal intensity projections to visualize the entire body of the LAA, given the frequent angulations and lobulations [66].

### 3.2. Three-Dimensional-Printed Models for Procedural Simulation

In the last decade, patient-specific 3D printing has represented an extension of CCT-based planning, enabling the transformation of volumetric imaging datasets into tangible anatomical models of the LAA. Through segmentation and 3D reconstruction, the complex geometry of the appendage can be accurately reproduced, providing a more comprehensive understanding of patient-specific anatomy than conventional two-dimensional imaging approaches [67,68,69] (Figure 6). The workflow generally includes image segmentation, three-dimensional reconstruction, mesh processing, and additive manufacturing. In LAAO applications, different software platforms have been used to generate patient-specific models from CCT datasets, including 3D Slicer [70], ITK-SNAP [71], AVIEW Modeler (Coreline Soft Incorporation, Republic of Korea) [72], and Mimics and 3-matics (Materialise, Belgium) [73]. The resulting models can then be exported as STL files and fabricated using different printing technologies and materials, according to the intended application. Reported LAAO models include FDM-printed P400 ABS (Stratasys, USA) [72], FDM-printed TPU 95A and SLA-printed Flexa693 photopolymer resin (DWS, Italy) [73], while more advanced procedural simulators have employed different materials, including Agilus30 (Stratasys, USA), Flexa Bright and Flexa Soft (Sinterit, Poland), to reproduce the interaction between cardiac tissues and procedural devices [74]. A major advantage of this technique is the ability to perform in vitro simulations of device deployment. Patient-specific physical models permit direct testing of different device sizes and identification of the optimal landing zone. This is particularly relevant given the marked anatomical heterogeneity of the LAA and the recognized limitations of standard sizing strategies, which may contribute to device mismatch and repeated deployment attempts. Consistently, the available evidence suggests that simulation-based planning may reduce the procedural duration, fluoroscopy exposure, and PDL rates associated with size selection [68,75].

The possibility of direct manipulation enhances spatial understanding and may assist in clarifying equivocal anatomical findings, thereby supporting procedural planning and, in selected cases, post-implant decision-making [76].

This approach is mainly based on the 3D printing of the isolated LAA anatomy, without including the whole anatomical scenario necessary to perform the entire LAAO procedure (LAA, left atrium, FO, superior and inferior vena cava, and femoral access). To overcome these limitations, recent patient-specific 3D models, including all these structures, have been incorporated into simulation platforms for procedural rehearsal as well as operator training, enabling reproduction of the key procedural steps within a controlled environment [74]. Figure 7 shows a representative patient-specific 3D-printed simulator designed for procedural rehearsal. The opened right atrium provides direct visualization of the FO, enabling simulation of TSP and LAA occluder implantation (Figure 7a). The implanted device is subsequently shown within the simulator and as it appears under fluoroscopic and TEE guidance (Figure 7b), demonstrating the ability of the model to realistically reproduce both procedural steps and multimodality imaging findings.

Despite these promising applications, several limitations remain. Current printing materials are unable to fully replicate tissue elasticity and the dynamic behavior of cardiac structures, potentially limiting the accuracy of simulated device-tissue interactions. In addition, the time, technical expertise, and costs associated with model production may limit widespread routine adoption, suggesting a preferential role for selected complex cases in which additional anatomical and procedural information is expected to provide the greatest benefit [72,73].

## 4. CT for Post-Procedural Follow-Up

Reliable imaging follow-up is essential to confirm procedural efficacy and to identify complications that may influence long-term outcomes [46]. In this context, CCT has gained increasing clinical relevance [14,77].

As in pre-procedural LAAO imaging, CT scanners with at least 64 slices are required, although 128-slice or dual-source systems are preferred for improved temporal resolution, image quality, and reduced radiation exposure [48,78]. High-quality acquisition is achieved with a temporal resolution up to 66 ms and a spatial resolution of approximately 0.5 mm. ECG-gated acquisition is essential, with prospective triggering preferred to minimize radiation dose. The standard contrast protocol involves biphasic injection of iodinated contrast (350–370 mg/mL) at 5–7 mL/s, with acquisition initiated at approximately 100–150 Hounsfield units (HU) in the left atrium or ascending aorta [48,78]. Delayed-phase imaging is not routinely performed during follow-up, as its clinical utility remains uncertain [79].

Follow-up imaging is often performed at 3 to 6 months and at 12 months, depending on institutional protocols and patient risk profile [80]. This interval allows assessment of:

- Device position and stability;
- DRT;
- PDL and LAA patency.

Figure 8 depicts examples of possible outcomes with and without complications at CCT follow-up imaging.

Beyond the first year, routine imaging is not universally required but may be considered in selected patients at higher risk of late complications [78,80].

### 4.1. Device Position and Stability

CCT provides a comprehensive and reproducible assessment of device position, stability, and orientation within the left atrium and enables precise visualization of the implant in relation to adjacent cardiac structures [78].

Beyond qualitative assessment of device alignment, CCT allows for the quantitative evaluation of implantation depth, measured from the left superior pulmonary vein ridge to the atrial surface of the device. A depth >10 mm has been associated with an increased risk of DRT, likely due to altered local flow dynamics and blood stasis within a recessed implantation zone [81]. These measurements, together with the assessment of device compression and apposition, provide indirect but robust markers of stable anchoring and adequate endothelialization.

### 4.2. Device-Related Thrombus

DRT, characterized by thrombus formation on the atrial surface of the device, has an incidence of about 3% [82]. This event is clinically significant, being strongly associated with thromboembolic events, including stroke and systemic embolism [83,84,85].

On TEE, DRT is defined as an independently mobile echo density in contact with the atrial surface of the device not attributable to artifact or normal healing. To improve diagnostic consistency, expert consensus recommends confirmation in multiple imaging planes and based on characteristic morphology (e.g., sessile or pedunculated appearance) [86]. However, diagnosis remains challenging, with substantial interobserver variability (~28% discordance), limited specificity in distinguishing thrombus from neointimal coverage or layered thrombus, and reduced sensitivity for thrombus located beneath the device membrane [86,87].

CCT has demonstrated superior sensitivity for detecting subtle surface abnormalities [88,89]. Thrombus is identified as hypoattenuated thickening (HAT), appearing as a low-density mass on the atrial surface of the device. However, interpretation requires caution to avoid false positives from normal anatomical or device-related structures, such as tissue invagination or the “screw hub cove,” which may mimic thrombus and require procedural correlation.

To standardize clinical decision-making, CCT-detected HAT is classified into grades 0 through 3 based on its thickness and morphology. Grade 0, or subfabric HAT, is unique to Watchman devices and refers to isolated low density within the cove located beneath the device fabric, representing intra-device thrombosis rather than surface thrombus. Grade 1 HAT is defined by a thickness ≤3 mm, or >3 mm if the surface is smooth and maintains continuity with the atrial wall, and it is generally viewed as a precursor to healthy endothelialization that does not require treatment escalation. Grade 2 HAT is characterized by a thickness >3 mm accompanied by an irregular surface or a lack of continuity with the atrial wall, while Grade 3 HAT refers to any pedunculated mass, regardless of size, on the device surface. Clinically, low-grade HAT (Grades 0–1) is considered benign, while high-grade HAT (Grades 2–3) is managed as definitive DRT [78,86,89].

Beyond ischemic stroke, systemic or peripheral embolization of DRT, intra-procedural thrombi, or air may result in ischemic complications involving other vascular territories, including acute limb ischemia, mesenteric ischemia, or renal infarction [83]. Although these events are rare and are not systematically reported as separate endpoints in large LAAO registries, they represent clinically relevant potential complications. Procedural vascular complications may also occur in relation to femoral access, including access-site hematomas, pseudoaneurysms, and arteriovenous fistulas.

### 4.3. Peri-Device Leak and LAA Patency

PDL refers to residual communication between the LA and the LAA after occlusion due to a non-complete sealing of the LAA. Although TEE remains the most widely used modality for post-procedural surveillance and allows direct comparison with intraprocedural imaging, its performance for PDL detection is limited by previously described technical and operator-dependent factors, as well as intrinsic blind spots that may lead to incomplete evaluation [47,78,90]. CT has therefore emerged as a superior imaging modality, providing a more comprehensive and reproducible assessment of peri-device sealing in the follow-up [47,91].

A key CCT-derived marker is LAA patency, defined as contrast opacification distal to the occlusion device in the absence of a demonstrable jet. This is typically quantified by distal attenuation >100 HU or an LAA-to-LA attenuation ratio ≥0.25 [92,93]. LAA patency may reflect either true residual peri-device flow (PDL) or delayed contrast permeation through device material (“fabric leak”), potentially indicating incomplete endothelialization [92,94,95].

CCT consistently shows a higher overall detection rate of PDL compared with TEE [90,91,95,96,97,98], although no difference has been reported for clinically relevant leaks (>5 mm) [77], a threshold originally defined arbitrarily in early device trials [77,99,100]. CCT is, however, more sensitive for smaller leaks (<5 mm) [100]. Emerging evidence suggests that even small PDLs may be associated with increased thromboembolic risk [101,102], and early post-procedural LAA patency with visible PDL has been linked to adverse outcomes [103,104]. CCT is uniquely capable of identifying the underlying mechanisms of these leaks, such as device malalignment, undersizing, or residual flow into uncovered accessory lobes.

## 5. Emerging Technologies and New Perspectives in LAAO

To date, CCT is the imaging modality that is best suited to support the planning and follow-up of the LAAO procedure. Over the past decade, advances in numerical simulations, artificial intelligence, and CT technology have further expanded its potential, opening new perspectives in the field of LAAO.

### 5.1. Numerical Simulations

In the context of computational modeling, two complementary approaches have emerged covering the two domains involved in the LAAO procedure: computational fluid dynamics (CFD) for the hemodynamics evaluation and finite element (FE) modeling for the structural analysis. Together, they provide a multi-scale framework to investigate both hemodynamic behavior and device-tissue interaction in LAAO. These models can provide insights into the understanding of main contributors of Virchow’s triad for the evaluation of thrombus formation in the LAA pre- and post-intervention.

#### 5.1.1. CFD for Hemodynamics and Thrombotic Risk

CFD simulations enable quantitative characterization of complex flow patterns through parameters such as velocity fields, wall shear stress (WSS), oscillatory shear index (OSI), and derived indices including endothelial cell activation potential (ECAP) and relative residence time (RRT), which collectively describe thrombogenic flow conditions [105,106,107,108,109].

Across studies, a consistent hemodynamic pattern emerges, characterized by progressive flow deceleration and WSS reduction from the LAA ostium toward distal lobes, where prolonged residence time and disturbed shear conditions promote blood stasis. Morphological determinants, such as enlarged LAA volume, a wide orifice, multilobed anatomy, and reduced contractility, play a central role in modulating intra-atrial flow, while pulmonary vein inflow further influences global atrial flow organization [40,110,111,112,113].

Recent developments have incorporated dynamic cardiac motion into CFD frameworks. Moving-wall models derived from ECG-gated or multiphase CT datasets provide more physiologically realistic flow patterns compared with rigid-wall assumptions, particularly in low-velocity and recirculation regions [114,115,116,117,118].

CFD has also been extensively applied to simulate the hemodynamic impact of LAAO by reconstruction of the post-implant anatomy from the follow-up CT, by positioning a virtual device in the pre-operative CT-based geometry. In general, device implantation improves flow organization by reducing LAA stagnation; however, residual low-velocity regions may persist depending on device type, positioning, and implantation depth. In particular, off-axis deployment, excessive depth, or incomplete ridge coverage are associated with suboptimal hemodynamic profiles and potentially increased thrombogenic risk [119,120,121,122,123]. In order to increase the reliability of these numerical models as useful tools for a clinical perspective in terms of thrombus risk formation (pre-LAAO), planning optimization (pre-LAAO), and DRT and PDL risk (post-LAAO), significant effort is now devoted to their validation using specific experimental methods.

In this context, experimental validation using mock circulatory loops and particle image velocimetry applied to CT-derived phantoms has further strengthened the translational value of CFD models, enabling high-resolution benchmarking of simulated flow fields [124,125].

#### 5.1.2. FE Modeling and FE-Informed CFD

Patient-specific FE modeling complements CFD by focusing on the mechanical interaction between occlusion devices and patient-specific LAA anatomy. These simulations allow prediction of device deformation, radial compression, anchoring forces, and final deployed configuration, thereby providing quantitative insight into procedural feasibility and mechanical stability [126].

Patient-specific virtual implantation enables systematic pre-procedural evaluation of device size, deployment depth, and orientation. FE simulations have shown good agreement with post-procedural CT in terms of device shape, compression, and landing zone apposition, supporting their potential to improve device selection and reduce PDL risk [127,128]. The clinical translation of patient-specific FE modeling has also led to the development of commercially available platforms, such as FEops HEARTguide (FEops NV, Belgium), which enables virtual LAAO by simulating device deployment on patient-specific CCTA-derived anatomy [126].

Recent advances include improved modeling of nitinol frame mechanics and patient-specific tissue heterogeneity, which enhance the realism of device expansion and anchoring behavior in complex anatomies [129,130]. These improvements are particularly relevant for accurately capturing sealing performance and implantation stability. More recently, FE modeling has been integrated with CFD to provide a more comprehensive assessment of post-implant hemodynamics, demonstrating the potential of FE-informed CFD to account for patient-specific device-tissue interaction when assessing post-LAAO flow patterns and thrombogenic risk [131].

Despite these advances, limitations remain, including high computational cost, uncertainties in tissue material properties, and variability in boundary conditions. Continued improvements in imaging-derived parameterization and computational efficiency are expected to facilitate broader clinical translation. Figure 9 shows the Virchow’s triad scheme with inclusion of numerical simulations, both CFD and FEA, as potential tools to detect the main factor categories contributing to thrombus formation.

### 5.2. Artificial Intelligence and Machine Learning

AI and machine learning (ML) are increasingly being integrated into the LAAO workflow, supporting risk stratification, imaging analysis, and procedural planning through data-driven approaches that complement traditional imaging interpretation (Figure 10).

Radiomics-based CT angiography analyses have demonstrated the ability to predict LAA hypercoagulability in AF, with radiomic signatures achieving strong discrimination performance and robust validation when combined with clinical variables [132]. More broadly, supervised ML models—including support vector machines, random forests, and gradient boosting algorithms—have shown superior performance compared with conventional regression models in multimodal thromboembolic risk prediction, while unsupervised methods enable identification of distinct phenotypic clusters of atrial disease [133].

Deep learning approaches have further advanced automated CT analysis, enabling robust segmentation of the left atrium and LAA across cardiac phases using convolutional neural network architectures. These methods allow extraction of functional and morphological parameters with high reproducibility, significantly reducing manual workload while maintaining diagnostic accuracy [27,134].

In the procedural domain, AI-assisted LAAO planning integrating CT imaging and intracardiac echocardiography has demonstrated procedural efficiency comparable to standard TEE-guided workflows, with potential reductions in device recapture and optimization of implantation strategy [26]. Additionally, explainable deep learning frameworks have been developed to identify imaging-derived predictors of thrombus formation, improving interpretability and clinical relevance through attention-based visualization techniques [135,136].

Despite these promising developments, current limitations include limited external validation, dataset heterogeneity, and reduced generalizability across institutions. The standardization of imaging pipelines and multicenter validation remain essential for the clinical translation of AI-based tools in LAAO [137,138].

### 5.3. Photon-Counting CT

Photon-counting computed tomography (PCCT) represents one of the most relevant recent advances in CT imaging. Unlike conventional energy-integrating detectors, photon-counting detectors directly convert individual X-ray photons into electrical signals and record their energy, enabling higher spatial resolution, improved noise performance, and intrinsic spectral imaging capabilities [139,140]. These benefits may be particularly relevant in the setting of LAAO.

In the pre-procedural setting, the enhanced spatial resolution of PCCT may facilitate more detailed characterization of LAA morphology, including complex lobar anatomy, trabeculations, and landing-zone dimensions, potentially supporting device selection and procedural planning.

The potential role of PCCT is also relevant for post-procedural assessment, in which metallic components of LAAO devices and concomitant calcifications can impair image interpretation because of blooming and beam-hardening artifacts. Recent clinical experience has specifically explored the application of ultra-high-resolution PCCT in patients with WATCHMAN devices. Diamant et al. reported four cases in which PCCT enabled the visualization of different post-implant findings, including fabric-deficiency leaks, small peri-device leaks, central leaks, and low-volume residual intra-device contrast ingress [141]. These observations highlight the potential of ultra-high-resolution PCCT for characterizing the device-tissue interface and detecting subtle residual communications following LAAO.

Beyond spatial resolution, the spectral capabilities of photon-counting technology may provide additional information through energy-resolved and material-specific reconstructions [142], potentially improving contrast visualization and the characterization of findings around implanted devices. These capabilities may also be relevant to the assessment of device-related thrombus and other post-procedural abnormalities. However, the current evidence remains limited, and the incremental diagnostic value of PCCT over conventional CCT for LAAO assessment has not yet been established. Larger prospective studies are therefore warranted to determine whether the technical advantages of PCCT translate into clinically meaningful improvements in pre-procedural planning and post-procedural surveillance.

## 6. Conclusions

The clinical management of AF has been significantly transformed by the integration of CCT into the LAAO workflow. By offering superior 3D anatomical detail and isotropic spatial resolution compared to traditional two-dimensional techniques, CCT has established itself as an essential tool for pre-procedural planning, allowing for precise device sizing and the identification of optimal procedural strategies.

In the post-procedural phase, CCT provides a comprehensive and reproducible assessment of device stability, PDL and DRT. The ability of CCT to identify even subtle leaks and surface changes ensures a high standard of follow-up care, which is critical for guiding antithrombotic therapy decisions.

Furthermore, the emergence of 3D printing, CFD, and AI represents a new frontier in personalized cardiac intervention. These advanced engineering and AI-driven tools enable patient-specific simulations of device-tissue interaction and hemodynamic behavior, paving the way for further procedural optimization and improved clinical outcomes. As these technologies continue to evolve and undergo multicenter validation, the role of CCT is expected to expand, reinforcing its position at the center of modern LAAO workflows.

## Figures and Tables

**Figure 1:**
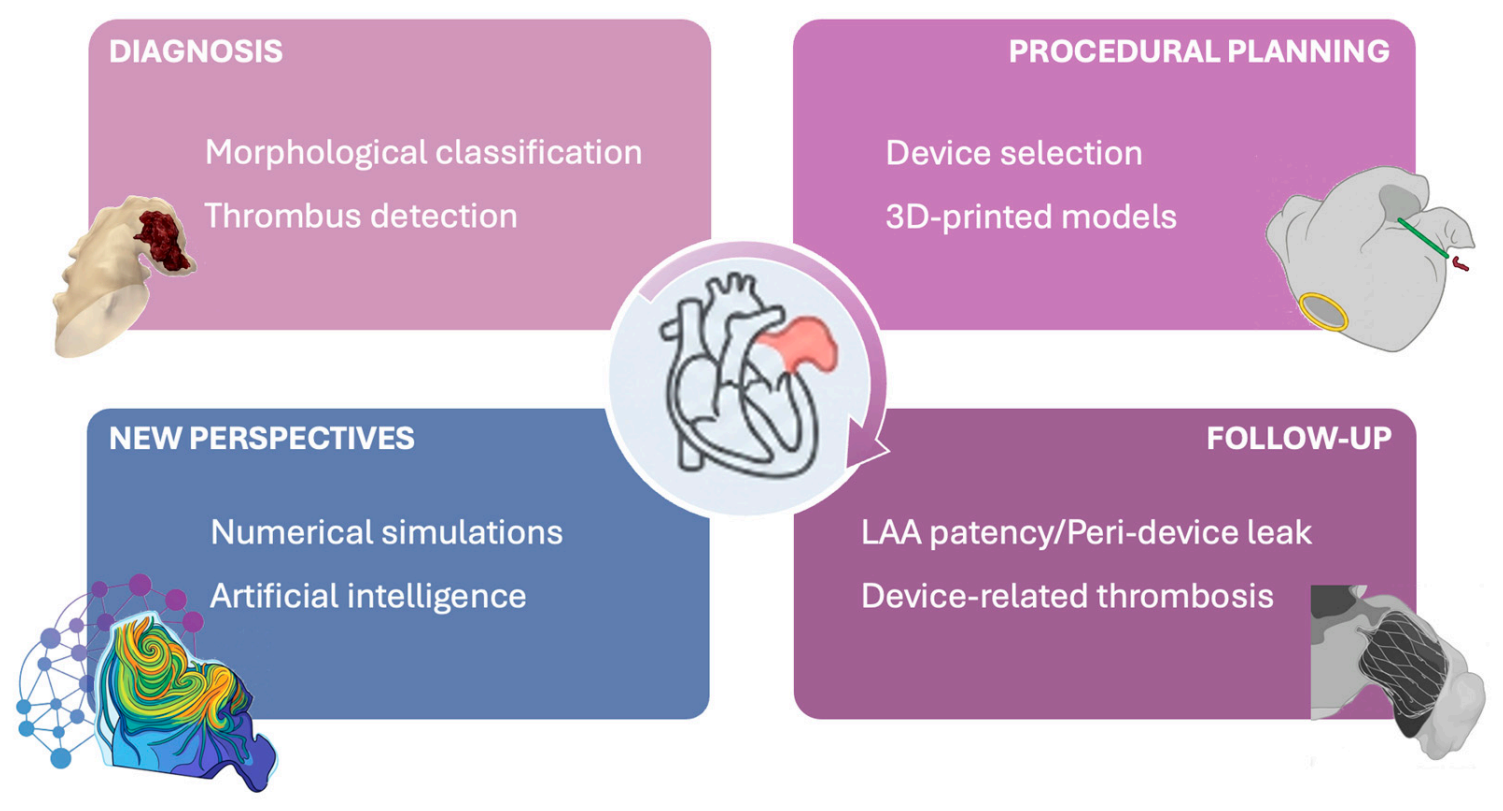
Representative schematic overview of the main phases of LAA management using CCT, including diagnosis, procedural planning, follow-up, and emerging perspectives. LAA = left atrial appendage, CCT = cardiac computed tomography.

**Figure 2:**
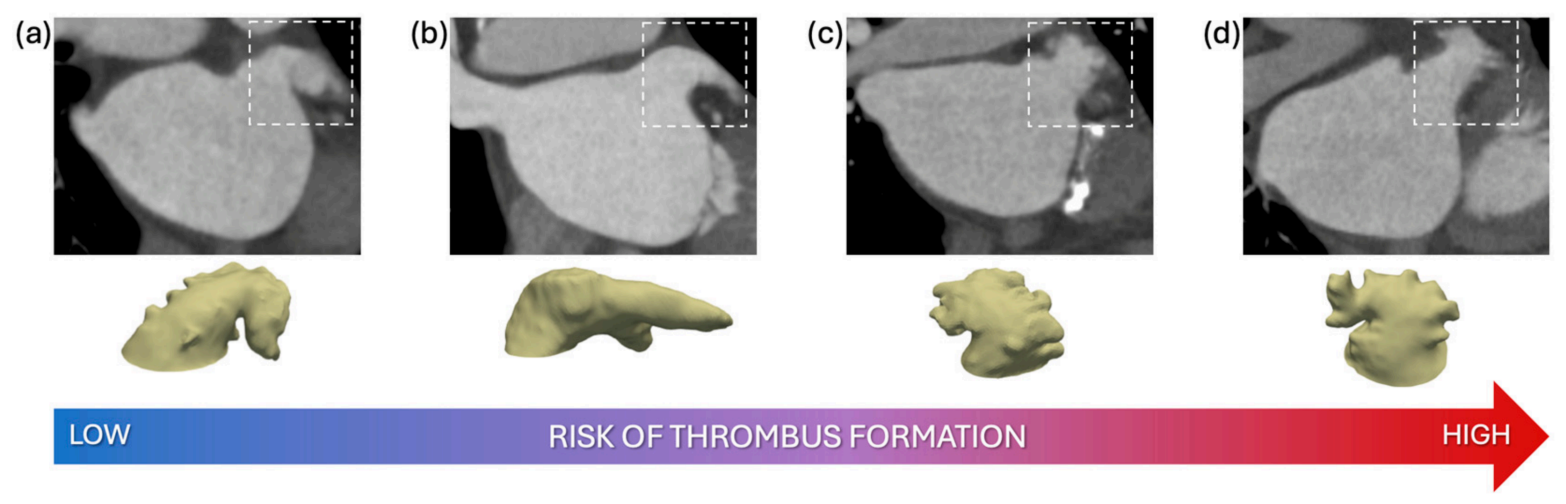
Example of the four morphologies of the LAA, with the associated risk of thrombus formation, according to Di Biase et al. [28]: chicken wing (**a**); cactus (**b**); windsock (**c**); cauliflower (**d**).

**Figure 3:**
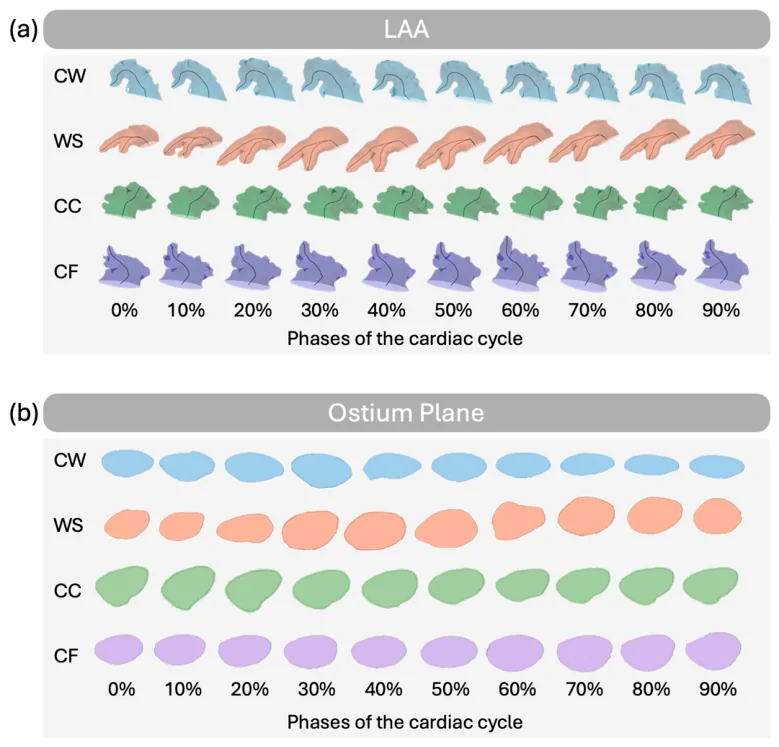
Example of LAA (**a**) and associated ostium variation (**b**) along the cardiac cycle, analyzed with the cardiac computed tomography for each morphological class. LAA = left atrial appendage, CW = chicken wing, WS = windsock, CC = cactus, CF = cauliflower.

**Figure 4:**
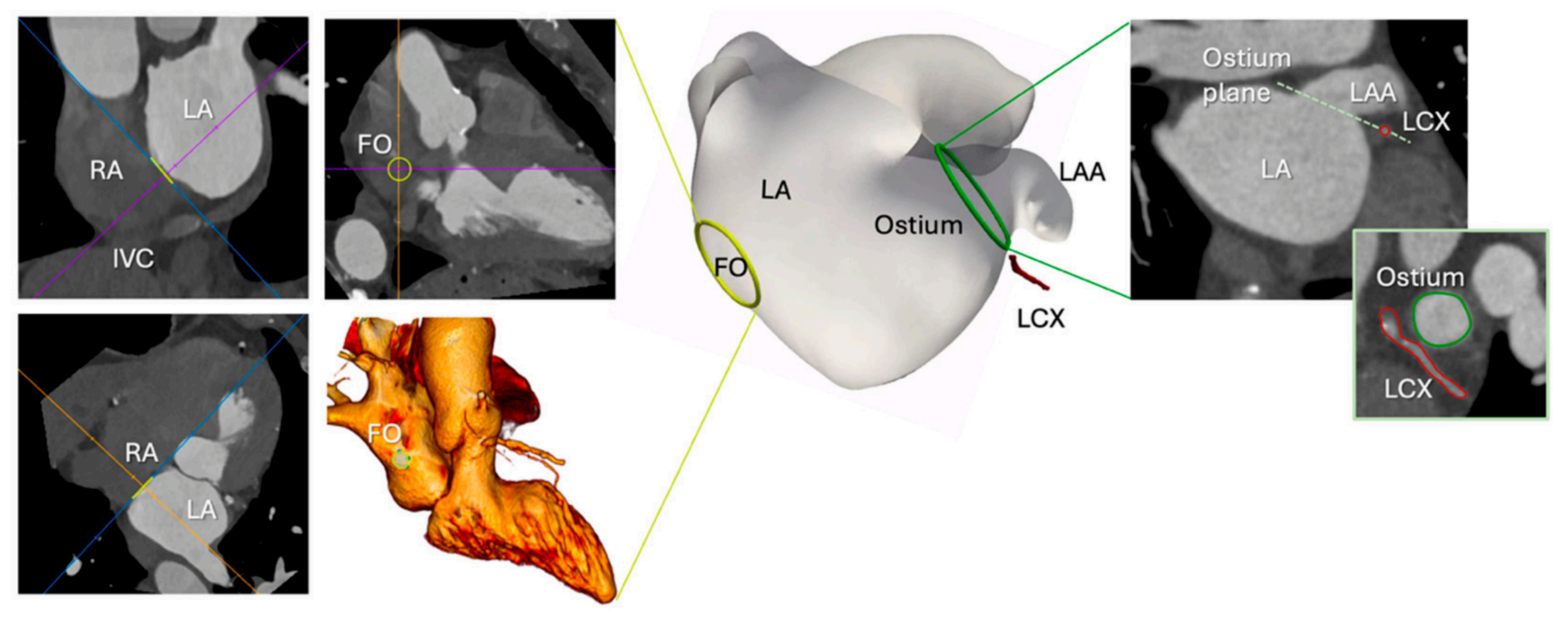
Example of LA and LAA from multiplanar reconstruction CCT images with indication of FO, ostium and LCX for pre-procedural planning. CCT = cardiac computed tomography, FO = fossa ovalis, LCX = left circumflex coronary artery, LA = left atrium, LAA = left atrial appendage, RA = right atrium, IVC = inferior vena cava.

**Figure 5:**
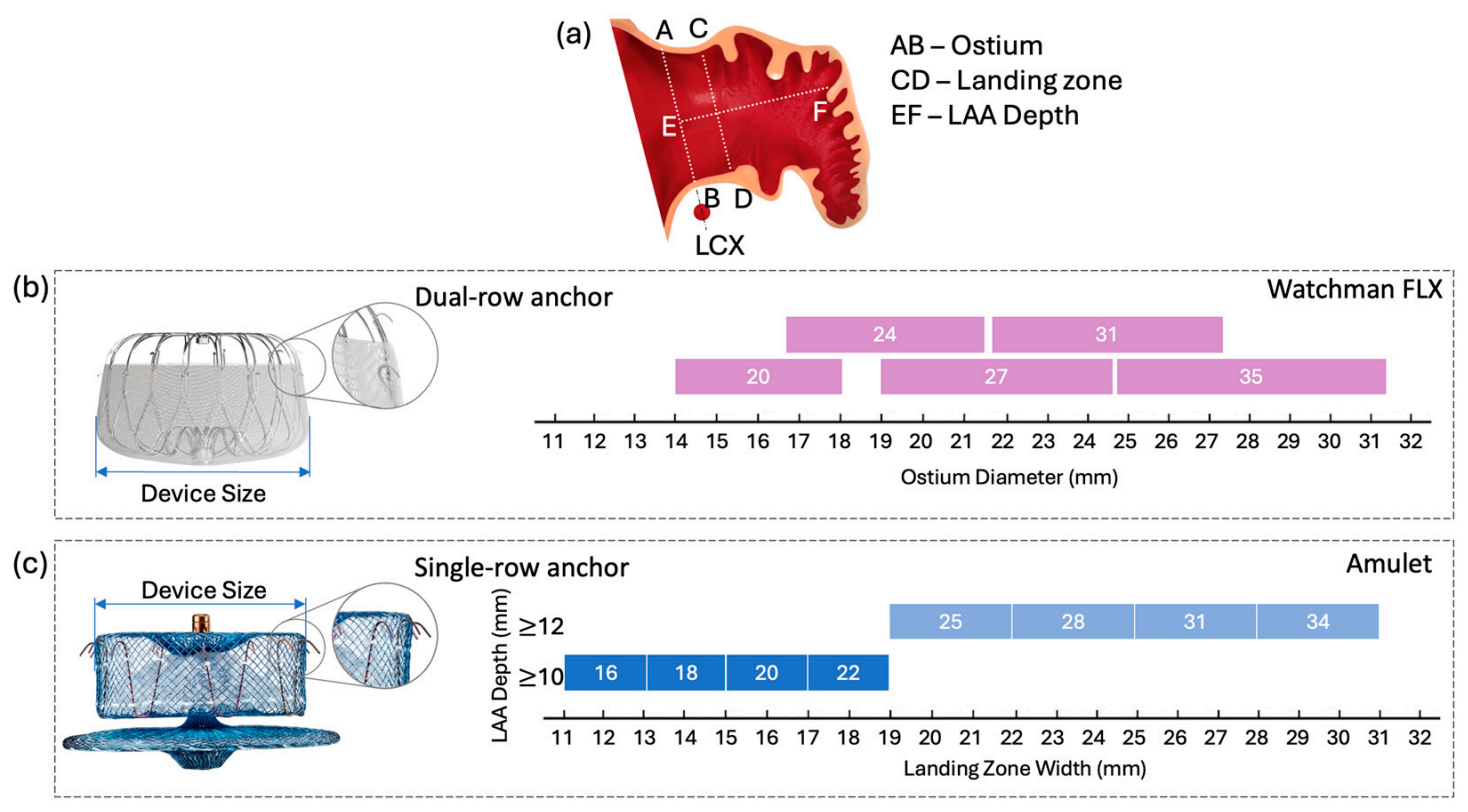
Representative LAA with indication of the main anatomical landmarks used for device selection and sizing definition (**a**). Example of Watchman FLX (**b**) and Amulet Amplatzer (**c**) with corresponding sizing charts. LAA = left atrial appendage, LCX = left circumflex coronary artery.

**Figure 6:**
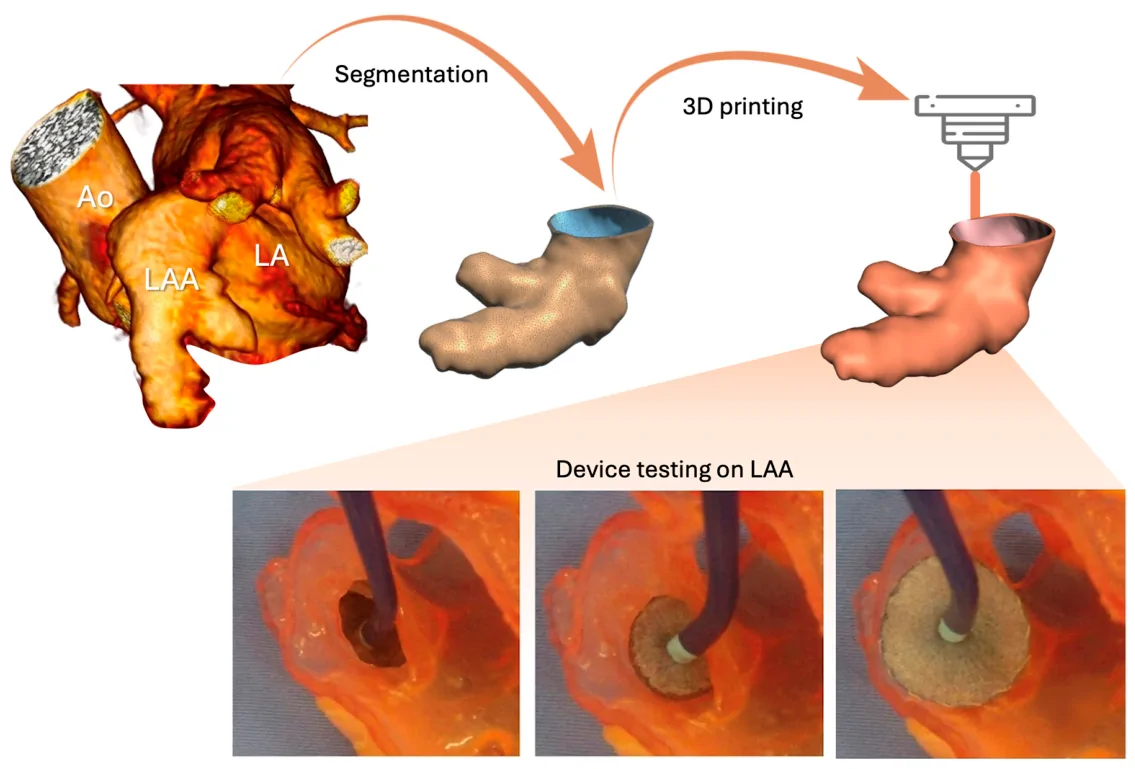
Representative workflow from the CCT to the 3D-printed LAA model used for the procedural simulation. CCT = cardiac computed tomography, Ao = aorta, LAA = left atrial appendage, LA = left atrium.

**Figure 7:**
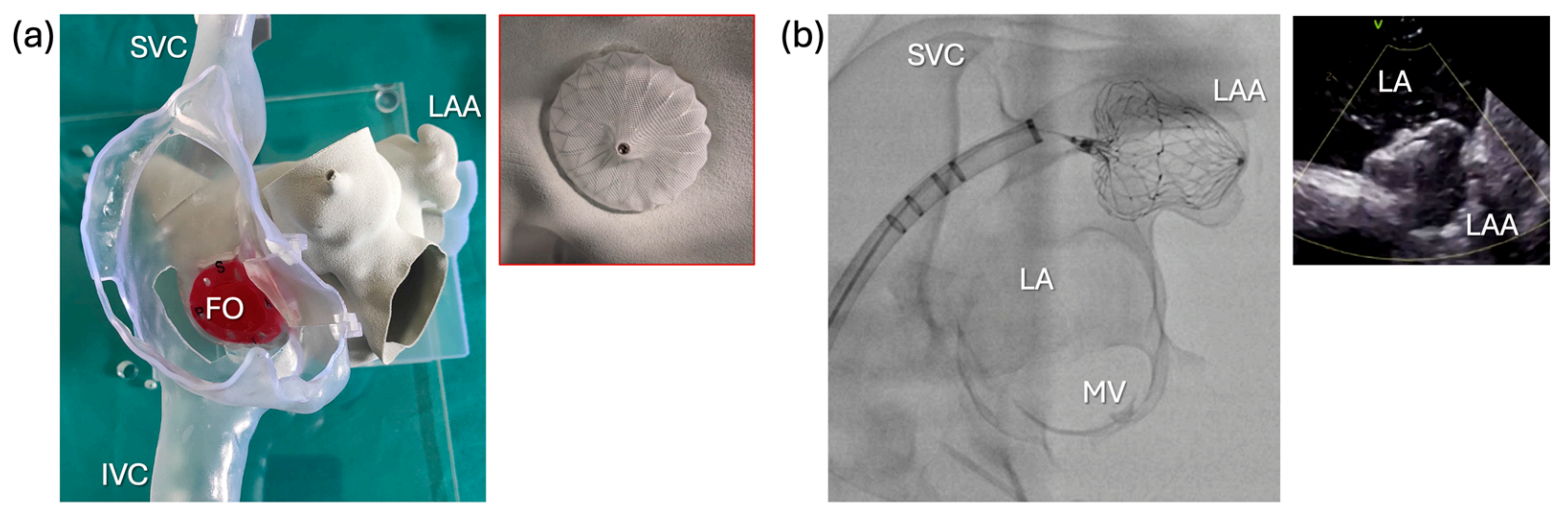
Example of patient-specific 3D-printed simulator used for the procedural simulation of the entire LAAO procedure (**a**). Example of intraoperative visualization of the simulator under fluoroscopic and trans-esophageal echocardiographic images (**b**). SVC = superior vena cava, LAA = left atrial appendage, IVC = inferior vena cava, FO = fossa ovalis, LA = left atrium, MV = mitral valve.

**Figure 8:**
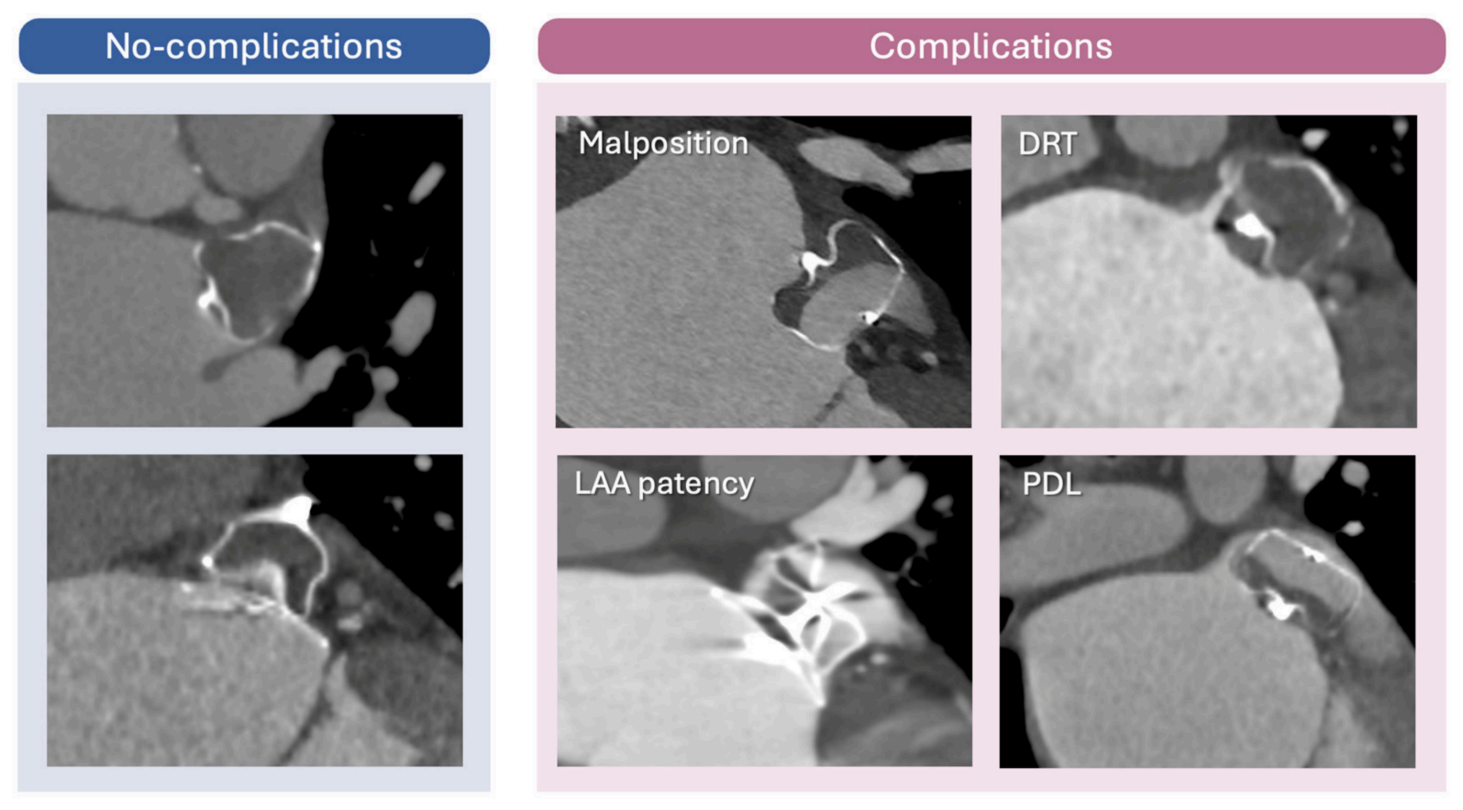
Example of CCT in the case of LAAO optimal outcomes and in the case of complications: malposition, DRT, LAA patency and PDL. DRT = device-related thrombus, LAAO = left atrial appendage, PDL = peri-device leak.

**Figure 9:**
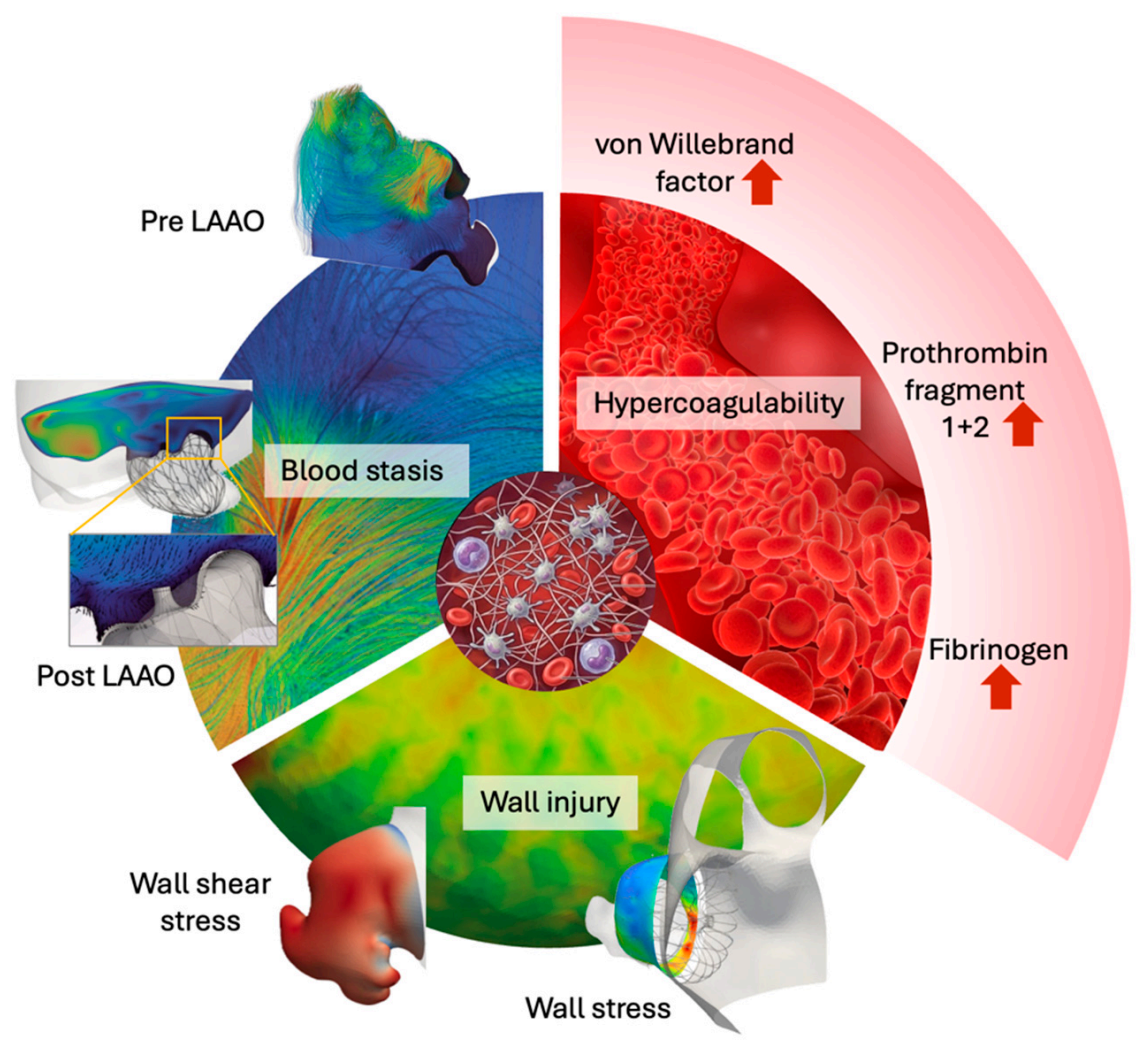
Scheme of the Virchow’s triad for the estimation with the three main categories of factors contributing to LAA and LAAO thrombus risk formation. LAAO = left atrial appendage occlusion.

**Figure 10:**
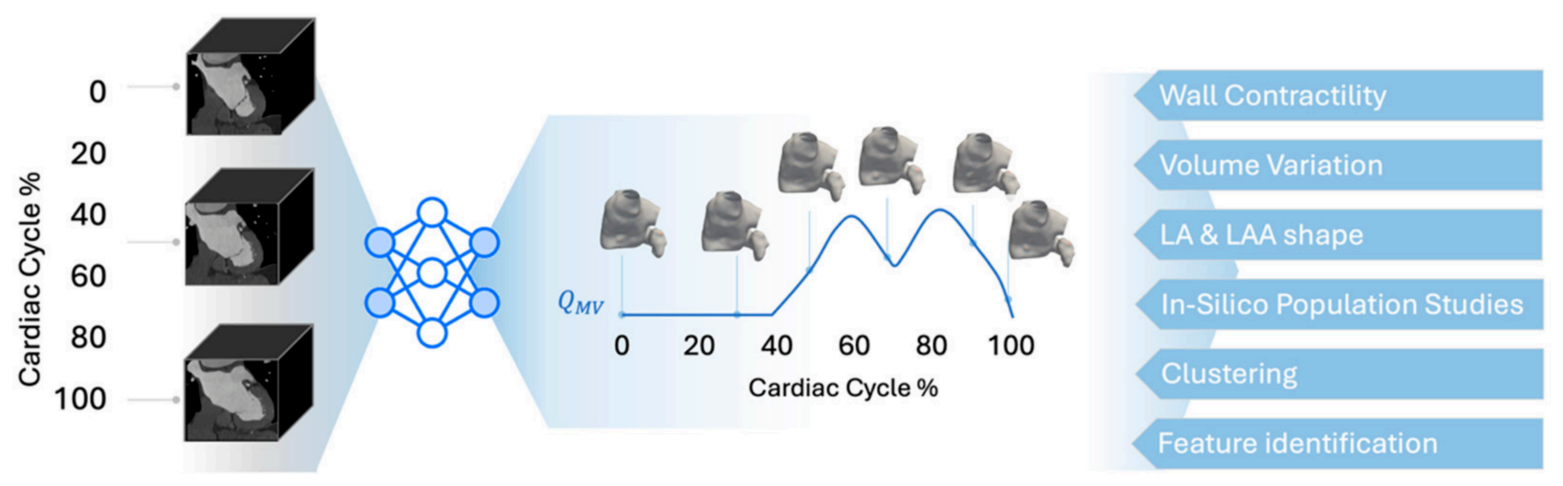
Example of CCT-based AI workflow for automatic extraction and classification of anatomical and functional time-variant features.LA = left atrium, LAA = left atrial appendage.

## Data Availability

No new data were created or analyzed in this study. Data sharing is not applicable to this article.
